# A Call to Improve a Chain of Cardiovascular Disease Care

**DOI:** 10.3389/ijph.2022.1605219

**Published:** 2022-08-24

**Authors:** Lei Hou

**Affiliations:** National Center for Chronic and Noncommunicable Disease Control and Prevention, Chinese Center for Disease Control and Prevention, Beijing, China

**Keywords:** cardiovascular disease, chain, out-of-hospital, hypertension, cardiac arrest

**FIGURE 1:**
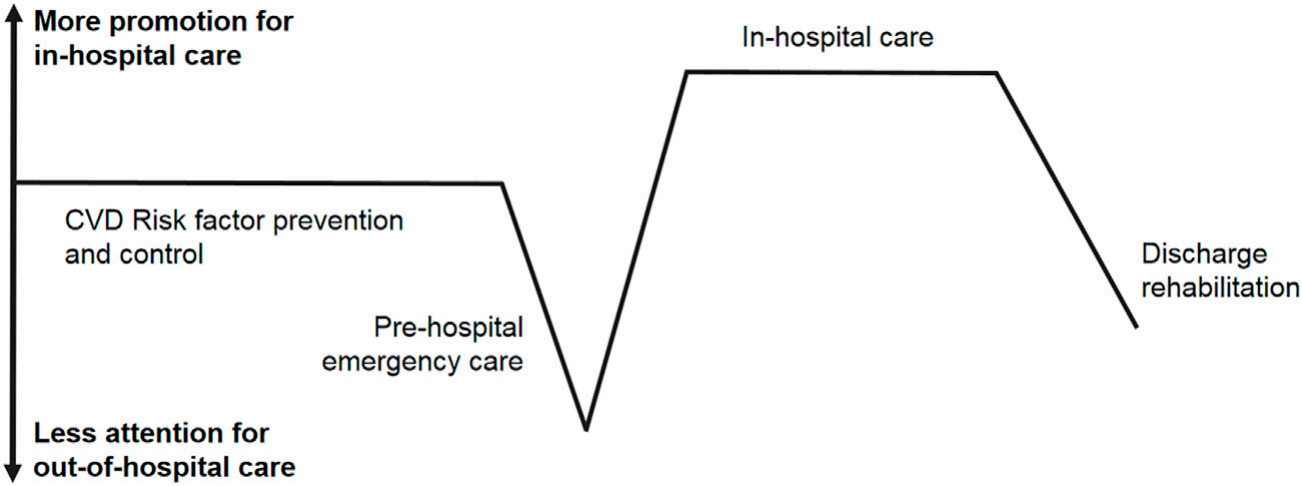
Current status of cardiovascular diseases prevention and control in China indicated by a chain of cardiovascular diseases care (Beijing, China. 2022). Note: CVD means cardiovascular diseases.

Cardiovascular disease (CVD), including stroke and acute myocardial infarction (AMI), is a leading cause of death in both urban and rural China (44.26% and 46.74% of total deaths) [1]. However, CVD mortality has been greater in rural than in urban areas in the last 10 years (up to 323.29 vs. 277.92 per 100,000 people) [1]. Reducing mortality from CVD in China therefore probably requires improvements in the chain of CVD care— a new concept (see Figure 1), including prevention, control, and treatment of both risk factors and disease. This chain will guide us to understand urban-rural differences, improve health services, and thereafter reduce CVD mortality in a broad perspective across the whole population, rather than simply hospital-based patients.

Hypertension accounts for 72.8% of the disease burden from stroke and 64.5% from other CVDs such as AMI and hypertensive heart disease, and is the most important risk factor for CVD [2]. There is a similar prevalence between urban and rural adult Chinese people (23.4% vs. 23.1%), but rural hypertensive patients have a worse rate of blood pressure control than urban patients (13.1% vs. 19.4%) [3]. This is despite improvements since 2012, when the overall rate in China was just 9.7%. These changes are probably associated with the National Primary Public Health Services (NPPHS) program of China, which was launched in 2009. Under this program, community doctors provide various free primary healthcare services for all registered citizens of China, including hypertension management. To date, the NPPHS has managed 100 million hypertensive patients, and this figure is expected to increase to 110 million by 2025. For most of the rural population, the main treatment for CVD is therefore village doctor-led usual care. A study has suggested that interventions led by village doctors, including both multifaceted interventions and enhanced usual care, result in significant improvements in blood pressure control among rural residents in China [4]. However, in less urbanized areas, drug use is still unsatisfactory in primary healthcare [5].

In the WHO MONICA Project, around 70% of patients with acute coronary heart disease died before reaching hospital. After over 30 years, this is still true in China. Only 18.9% of CVD deaths currently happen in hospital, and this falls to 14.0% in rural China [6]. This may be because of poorer emergency medical services in rural areas. Out-of-hospital cardiac arrest, which has an extremely low survival rate (generally lower than 1%) and contributes to half of heart deaths, keeps challenging the CVD care chain [7]. Ongoing improvements are related to bystander cardiopulmonary resuscitation (CPR), advanced life support provided by emergency medical services, and in-hospital care. Provision of basic life support by non-professional bystanders increases the survival chance of people who have a cardiac arrest outside hospitals. However, CPR training for the public and public provision of automatic external defibrillators are both insufficient.

In-hospital CVD care is generally better than other links of the chain. China has focused on improving in-hospital care quality. Recently, there have been several studies exploring differences in in-hospital mortality from AMI between urban and rural regions [8–10]. One study in particular suggested that the difference could be reduced by percutaneous coronary intervention (PCI) [10], but this is unlikely to be the only relevant factor. Urban and rural areas have different levels of social and economic development. This inevitably leads to divergence in population characteristics, demand for healthcare, provision of specific care, and patients screened by the chain of CVD care. The divergence is also linked to availability and effect of PCI. The urban–rural differences related to in-hospital mortality from AMI reflect all these factors, and not just a single intermediate factor such as PCI, but it remains important to enhance primary healthcare clinics and hospitals, especially in developing rural regions. Additionally, hierarchical analyses exploring the impact of particular factors such as PCI on the association between exposure and disease are indispensable to the development of a complex and conditioned statistical model adjusting for multiple factors.

CVD discharge rehabilitation needs more attention. China currently has 13 million people with stroke and 11 million with coronary heart disease. However, there is little high-quality research evidence to support the development of policies for rehabilitation after stroke and myocardial infarction. In rural regions, these patients may be more dependent on blood pressure and blood sugar management from the NPPHS than those living in cities where there are more medical resources.

In conclusion, out-of-hospital CVD care is the main area that needs attention in this care chain. Out-of-hospital links, including risk factor control, pre-hospital emergency care, and discharge rehabilitation, are much weaker than in-hospital care in the overall chain of CVD care. The difference between rural and urban regions is systemic, and both out-of-hospital and in-hospital care need to be enhanced in rural areas. However, the most cost-effective course of action in both urban and rural areas is undoubtedly to be controlling CVD risk factors such as hypertension, smoking, unhealthy diet, and lacking physical activity.
